# Editorial: Interactive Digital Technologies and Early Childhood

**DOI:** 10.3389/fpsyg.2019.02764

**Published:** 2019-12-11

**Authors:** Jennifer L. Miller, Kathleen A. Paciga, Carly A. Kocurek, Arlen Moller

**Affiliations:** ^1^Department of Psychology, Illinois Institute of Technology, Chicago, IL, United States; ^2^Humanities, History, and Social Sciences, Columbia College Chicago, Chicago, IL, United States; ^3^Department of Humanities, Illinois Institute of Technology, Chicago, IL, United States

**Keywords:** digital technologies, child development, interactive, early childhood, technology

This special Research Topic sought to increase our understanding of how interactive digital technologies (e.g., mobile devices, smartphones, tablets, robotics) impact learning and development in children from birth to 5 years. This topic is timely as there is considerable attention to the role that digital technologies play in young children's lives. A relatively emerging field called computer-child interactions (CCI), which seeks to understand the activities, behavior and abilities surrounding children's engagement with technologies, is becoming an area of great interest (Read and Bekker, 2011). There is a growing need to understand child-computer interactions, integration of digital technologies in early childhood education, and importantly, the role it plays on learning and development.

We received 22 proposals for the special issue, accepted eleven, and received five completed manuscripts, which are published in this special Research Topic. Of the five articles published in this Research Topic three were empirical studies, one was a meta-analysis, and one was a theoretical think piece (Sziron and Hildt).

All three of the empirical studies focused on child-computer interactions in different contexts. O'Byrne et al. examined the viability of an instructional model that leveraged digital tools in the composition process in a formal preschool setting. The remaining two empirical studies engaged children in more controlled laboratory settings for the research. Etta and Kirkorian explored the relation between the interactive features in the design of an eBook application and the effect of such features on 3- to 5-year olds' word learning and story comprehension. Finally, Martens et al. articulated a child-centered design approach demonstrating how children can serve as co-designers to develop an alphabet app. Taken together, these studies highlight the importance of utilizing digital technologies in early childhood education contexts and demonstrating the relation of design principles to outcomes. Finally, Xie et al. conducted a meta-analysis across 36 empirical articles to test the effect of touchscreen devices on learning and found a moderate effect size that young children do learn from touchscreens.

While the other articles provided empirical and quantitative support, Sziron and Hildt provide an elegant discussion of ethical issues related to children's use of interactive digital technologies. As an organizing principle, they use a “*Child's Right to an Open Future*,” which concerns tension between children's use of digital technology without having developed capacities to make informed choices about how use might positively or negatively influence children's social, emotional, and intellectual development. One facet explored involved the ethical responsibility to teach children to be informed and culturally responsible digital citizens, e.g., as part of compulsory public education. Another concerns respecting children's rights to privacy, including required parent's permission for processing data and children's “right to be forgotten” (i.e., to have personal data deleted). A final ethical consideration explored involved *justice* with respect to distributing the best digital active learning resources, and media designed with and for children.

We believe these articles included in this special issue are good examples of how digital technologies can play a role in children's lives. Taken together, these articles illustrate contexts in which (a) digital technologies can function in early childhood education contexts, (b) considerations for designing developmentally appropriate media for digital technologies, (c) that digital technologies can facilitate learning, and (d) the ethical considerations related to children's use of digital technologies. In addition to providing a forum for understanding the role of digital technologies during early childhood, our hope is that the field continues to examine these issues.

## Author Contributions

All authors listed have made a substantial, direct and intellectual contribution to the work, and approved it for publication.

### Conflict of Interest

The authors declare that the research was conducted in the absence of any commercial or financial relationships that could be construed as a potential conflict of interest.
